# Clinical and Inflammatory Determinants of Postoperative Delirium After Hip Fracture Surgery: A Retrospective Cohort Study of 962 Patients

**DOI:** 10.3390/medicina62081529

**Published:** 2026-08-09

**Authors:** Muhammed Kazez, Sevler Yıldız, Ümit Karatepe

**Affiliations:** 1 Department of Orthopaedics and Traumatology, Elazığ Fethi Sekin City Hospital, 23280 Elazığ, Türkiye; 2 Department of Psychiatry, University of Health Sciences, Elazığ Fethi Sekin City Hospital, 23280 Elazığ, Türkiye; dr_sevler@hotmail.com; 3 Department of Anesthesiology and Reanimation, Elazığ Fethi Sekin City Hospital, 23280 Elazığ, Türkiye; ukaratepe23@gmail.com

**Keywords:** hip fracture, postoperative delirium, C-reactive protein-to-albumin ratio, inflammation, hemiarthroplasty, proximal femoral nailing, risk factors

## Abstract

*Background and Objectives:* Postoperative delirium (POD) is a frequent neuropsychiatric complication following hip fracture surgery in older adults and is associated with prolonged hospitalization, functional decline, and increased mortality. Systemic inflammation is thought to play a central role in its pathogenesis, and the C-reactive protein CRP-to-albumin ratio (CAR) has emerged as a promising inflammatory biomarker. This study aimed to investigate the association between the preoperative CAR and POD and to identify clinical and perioperative factors independently associated with POD. *Materials and Methods:* This retrospective cohort study included 962 consecutive patients who underwent surgery for hip fractures between January 2022 and December 2025. Patients with femoral neck fractures underwent hemiarthroplasty, whereas those with intertrochanteric fractures were treated with proximal femoral nailing. Cases of postoperative delirium were evaluated following psychiatric consultation requested based on clinical suspicion by the orthopedic team, nursing staff or the patient’s caregiver; the definitive diagnosis was made by consultant psychiatrists according to the DSM-5 diagnostic criteria. Demographic characteristics, perioperative variables, and preoperative laboratory parameters were analyzed. Univariable and prespecified multivariable logistic regression analyses were performed to identify factors independently associated with POD. Model discrimination and calibration were assessed using receiver operating characteristic analysis, bootstrap internal validation, and the Hosmer–Lemeshow goodness-of-fit test. *Results:* Postoperative delirium developed in 112 of 962 patients (11.6%). Patients who developed POD had significantly higher preoperative CAR values than those without POD (0.76 vs. 0.33, *p* < 0.001). In the multivariable analysis, male sex (OR 1.567, 95% CI 1.007–2.438; *p* = 0.046), hemiarthroplasty (OR 1.931, 95% CI 1.213–3.075; *p* = 0.006), perioperative blood transfusion (OR 1.870, 95% CI 1.178–2.971; *p* = 0.008), general anesthesia (OR 1.829, 95% CI 1.093–3.061; *p* = 0.021), higher ASA physical status classification (OR 2.154, 95% CI 1.346–3.446; *p* = 0.001), longer time from hospital admission to surgery (OR 1.250 per 24 h increase, 95% CI 1.103–1.417; *p* < 0.001), and a higher log_2_-transformed CAR (OR 1.184 per doubling, 95% CI 1.037–1.352; *p* = 0.013) were independently associated with POD. The final model demonstrated acceptable discrimination (AUC 0.764; optimism-corrected AUC 0.744) and satisfactory calibration (Hosmer–Lemeshow *p* = 0.242). *Conclusions:* A higher preoperative C-reactive protein-to-albumin ratio was independently associated with postoperative delirium after hip fracture surgery, even after adjustment for established demographic and perioperative risk factors. Male sex, hemiarthroplasty, general anesthesia, higher ASA physical status classification, perioperative blood transfusion, and delayed surgery were also independently associated with POD. These findings suggest that CAR may be a low-cost and readily available inflammatory biomarker that could be used to develop a preoperative risk classification system for the development of postoperative delirium. However, since routine standardized delirium screening was not performed during the study, cases of hypoactive delirium in particular may not have been adequately identified. For this reason, the findings should be interpreted with caution. Prospective, multicenter studies incorporating standardized delirium screening methods are needed to validate these results and determine the additional predictive value of CAR beyond established clinical risk factors.

## 1. Introduction

Hip fractures are among the most common orthopedic injuries in older adults and are associated with substantial mortality, morbidity, and healthcare costs [1]. As life expectancy increases and the global elderly population continues to grow, the incidence of hip fractures is expected to rise even further in the coming decades [2]. These fractures typically result from low-energy trauma and are frequently associated with loss of functional independence, prolonged institutional care, and reduced quality of life in older adults [3].

Hip fractures are anatomically classified as intracapsular or extracapsular. Femoral neck fractures are intracapsular injuries with distinct biological and biomechanical characteristics, whereas intertrochanteric and subtrochanteric fractures constitute the extracapsular group [4,5,6]. Proximal femoral nailing (PFN) is currently the preferred treatment for most extracapsular hip fractures because it is a minimally invasive technique that provides stable fixation through smaller surgical incisions, minimizes soft tissue damage, and is generally associated with shorter operative times. However, depending on fracture characteristics and biological healing potential, immediate full weight-bearing may not always be feasible during the early postoperative period. In contrast, hemiarthroplasty (HA) is commonly performed for displaced femoral neck fractures in older adults. Unlike fracture fixation, HA restores hip function independently of fracture healing and generally permits immediate full weight-bearing. Nevertheless, it is a more invasive procedure requiring greater surgical exposure and is often associated with increased blood loss [7,8].

Among the postoperative complications following hip fracture surgery, postoperative delirium (POD) is one of the most common neuropsychiatric disorders in older adults and has a substantial impact on clinical outcomes [9,10,11,12]. Delirium is characterized by an acute, fluctuating disturbance in attention, awareness, and cognition and is associated with prolonged hospitalization, delayed functional recovery, increased dependency, and higher mortality rates [13,14,15]. The reported incidence of POD after hip fracture surgery ranges from 13% to 55%, depending on patient characteristics and the diagnostic methods used [16]. Numerous factors, including advanced age, dementia, comorbidities, malnutrition, infections, electrolyte imbalances, and delayed surgery, have been associated with the occurrence of POD [17,18]. Nevertheless, the pathophysiology of POD remains incompletely understood, and its early identification continues to represent an important clinical challenge.

In recent years, increasing attention has focused on the role of systemic inflammation and neuroinflammatory pathways in the development of POD [19,20,21,22]. The inflammatory response triggered by surgical trauma may increase blood–brain barrier permeability, activate microglia, and disrupt neurotransmitter homeostasis, ultimately leading to acute cerebral dysfunction [19,20,21]. Consequently, numerous inflammatory biomarkers have been investigated for their association with POD. Previous studies have reported significant associations between POD and inflammatory markers such as the neutrophil-to-lymphocyte ratio, platelet-to-lymphocyte ratio, and C-reactive protein (CRP) levels [23,24,25,26]. However, biomarkers that reflect both systemic inflammation and nutritional status may provide a more comprehensive assessment of the patient’s physiological condition.

The C-reactive protein-to-albumin ratio (CAR) is a simple, inexpensive, and readily available biomarker that simultaneously reflects systemic inflammatory burden and nutritional status [27]. Previous studies have suggested that elevated CAR levels are associated with postoperative delirium following total knee arthroplasty [28]. Similarly, studies involving older adults with hip fractures have demonstrated an association between elevated preoperative CAR levels and postoperative delirium, as well as other adverse clinical outcomes [29].

Identifying patients at high risk for postoperative delirium among those undergoing hip fracture surgery early on can facilitate the timely implementation of preventive strategies and contribute to the optimization of perioperative management [30]. However, there are limited studies that simultaneously evaluate inflammatory biomarkers, clinical characteristics, and perioperative factors within a comprehensive multivariate predictive model. Furthermore, there are very few studies that systematically examine the model’s discrimination, calibration, and internal validity while assessing the independent association of CAR. Accordingly, the primary objective of this study is to examine the association between preoperative CAR and postoperative delirium; the secondary objective is to identify other clinical factors independently associated with postoperative delirium. In our study, we hypothesized that high preoperative CAR values would continue to show an independent association with postoperative delirium even after adjusting for established demographic and perioperative risk factors.

## 2. Materials and Methods

### 2.1. Study Design and Patient Selection

This retrospective cohort study was conducted at the Department of Orthopaedics and Traumatology of a tertiary referral center in Türkiye. The study protocol was approved by the Firat University Non-Interventional Research Ethics Committee, Elazig, Türkiye (Approval No. 2024/03-73; 13 February 2024), and was conducted in accordance with the principles of the Declaration of Helsinki. This study was reported in accordance with the Strengthening the Reporting of Observational Studies in Epidemiology (STROBE) guidelines.

Medical records of consecutive patients who underwent surgical treatment for hip fractures between January 2022 and December 2025 were retrospectively reviewed. Patients aged 50 years or older who sustained hip fractures following low-energy trauma (simple falls) and had complete preoperative laboratory data were eligible for inclusion. According to the institutional treatment protocol, patients with displaced femoral neck fractures underwent hemiarthroplasty (HA), whereas those with intertrochanteric fractures were treated with proximal femoral nailing (PFN).

Patients with pathological fractures, high-energy trauma, preoperative delirium, revision surgery, intraoperative mechanical complications, or incomplete clinical or laboratory data were excluded. In addition, patients who required reoperation within the first postoperative month because of periprosthetic fracture, implant cut-out, hip or prosthetic dislocation, superficial or deep surgical site infection, or periprosthetic infection were excluded.

Patients requiring early reoperation were excluded from the study because major postoperative mechanical or infectious complications can significantly alter the postoperative clinical course, prolong the hospital stay, increase the systemic inflammatory response, and influence the development of postoperative delirium. These patients were excluded to minimize bias that could arise from serious postoperative complications developing after the index surgery and to obtain a more homogeneous study population.

**Figure 1:**
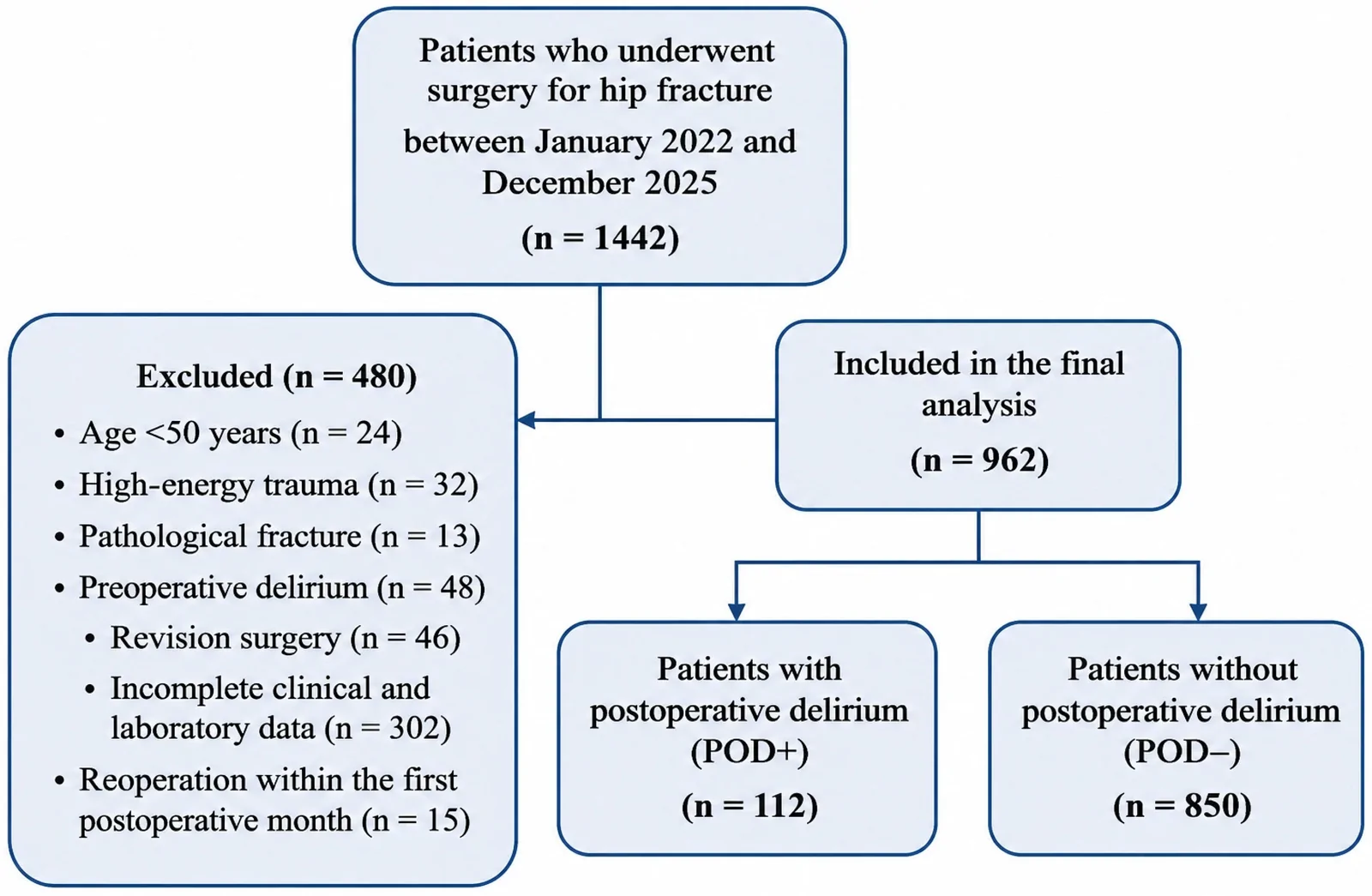
Flowchart of patient selection and study cohort formation. POD: postoperative delirium.

The study database was locked on 15 February 2026 following completion of data extraction and verification. After application of all inclusion and exclusion criteria, 962 eligible patients were included in the final analysis. The patient selection flowchart is shown in Figure 1.

### 2.2. Data Collection and Variables

Demographic, clinical, and laboratory data were retrospectively extracted from the hospital electronic medical record system and archived patient records. Demographic variables included age, sex, and body mass index (BMI). To facilitate clinically meaningful subgroup analyses, age categories were predefined based on the age groups commonly used in the literature on geriatric hip fractures [29,30].

Clinical variables included fracture side, surgical procedure (hemiarthroplasty [HA] or proximal femoral nailing [PFN]), American Society of Anesthesiologists (ASA) physical status classification, anesthesia type, time from hospital admission to surgery, operative time, perioperative blood transfusion, preoperative and postoperative mobilization status, perioperative complications, pulmonary embolism (PE), and deep vein thrombosis (DVT).

Comorbidity was defined as the presence of one or more of the following chronic conditions: hypertension, diabetes mellitus, coronary artery disease, chronic obstructive pulmonary disease, or chronic kidney disease.

Perioperative complications were defined as the occurrence of pneumonia, urinary tract infection, acute kidney injury, cardiac arrhythmia, or other systemic complications requiring additional treatment or close monitoring during hospitalization. Because pulmonary embolism and deep vein thrombosis were analyzed separately as postoperative thromboembolic complications, they were not included in the composite perioperative complication variable.

Information regarding dementia, preadmission psychoactive medication use, infection at hospital admission, and pre-fracture residence was available in the medical records. However, these variables were not included in the multivariable analysis because of incomplete or heterogeneous data. Baseline cognitive assessments, frailty indices, and formal nutritional assessment scores were not routinely available in the institutional database.

Preoperative laboratory parameters included hemoglobin (Hb), white blood cell count (WBC), platelet count (PLT), international normalized ratio (INR), sodium, potassium, chloride, calcium, aspartate aminotransferase (AST), alanine aminotransferase (ALT), urea, creatinine, total bilirubin, C-reactive protein (CRP), erythrocyte sedimentation rate (ESR), albumin, venous partial pressure of oxygen (PvO_2_), venous partial pressure of carbon dioxide (PvCO_2_), and lactate. All laboratory measurements were obtained from the first blood samples collected within 24 h before surgery. Venous blood gas analysis was routinely performed on admission to the emergency department before oxygen supplementation as part of the standard preoperative assessment.

The CAR was calculated by dividing the serum CRP concentration (mg/L) by the serum albumin concentration (g/L). Because the CAR distribution exhibited a pronounced right-skewed distribution, a log_2_ transformation was applied prior to regression analysis. Accordingly, the reported odds ratio represents the change in the likelihood of postoperative delirium associated with a twofold increase in the CAR value.

### 2.3. Definition and Assessment of Postoperative Delirium

POD has been defined as delirium that develops during the hospital stay following surgery and is confirmed by a consulting psychiatrist. When delirium was clinically suspected, a psychiatric consultation was requested by the on-call orthopedic surgeon. This suspicion was primarily based on nursing observations and reports from family members regarding sudden changes in consciousness, disorganized speech, or unusual behavior, as well as the orthopedic team’s clinical assessment. No formal delirium assessment algorithm or standardized screening tool was used during the study. Instead, patients exhibiting acute agitation, altered consciousness, disorganized speech, disorientation, or other behavioral changes suggestive of delirium were referred for psychiatric evaluation.

Delirium diagnoses were identified through retrospective review of psychiatric consultation records and electronic medical records. In all cases, the diagnosis was established according to the *Diagnostic and Statistical Manual of Mental Disorders*, Fifth Edition (DSM-5). For this reason, the primary endpoint of the study was postoperative delirium confirmed by a psychiatrist according to DSM-5 criteria during the hospital stay, rather than delirium detected through routine standardized screening.

Patients diagnosed with delirium were included in the POD (+) group, while patients in whom delirium was not detected were included in the POD (−) group. The timing of delirium onset was recorded from the electronic medical records, and patients were followed throughout hospitalization until discharge. Because no routine standardized delirium screening tool, such as the Confusion Assessment Method, was implemented, some cases, particularly hypoactive delirium, may have remained unrecognized.

### 2.4. Surgical Treatment and Perioperative Management

The surgical procedure was determined according to fracture type. Patients with displaced femoral neck fractures underwent hemiarthroplasty, whereas those with intertrochanteric fractures were treated with proximal femoral nailing (PFN) according to the institutional treatment protocol. The Smith & Nephew InterTAN^®^ (Memphis, TN, USA) intramedullary nail system was used in the PFN group, whereas the Tıpmed^®^ (Tıpmed Medical Device Manufacturing Company, İzmir, Türkiye) bipolar hemiarthroplasty system was used in the HA group.

All intertrochanteric fractures were treated using a standardized short Smith & Nephew InterTAN^®^ nail throughout the study period, without variation in implant design or nail length.

All hemiarthroplasty procedures were performed using the standard posterolateral approach. Throughout the study period, the standard treatment for displaced femoral neck fractures at our institution was cementless bipolar hemiarthroplasty. Thanks to the availability of a wide range of cementless femoral stem sizes, adequate primary press-fit fixation could be achieved even in patients with relatively wide femoral canals. For this reason, a wide femoral canal alone was not considered an indication for cemented fixation, and cemented femoral stems were not routinely used.

All surgical procedures were performed by the same orthopedic surgery team, and both surgical techniques and perioperative management protocols remained consistent throughout the study period.

Operative time was defined as the interval between skin incision and wound closure. Time to surgery was defined as the interval between hospital admission and the start of surgery.

Perioperative management was standardized according to the institutional treatment protocol. Patients underwent either spinal or general anesthesia according to the clinical judgment of the attending anesthesiologist and individual perioperative requirements. Intravenous cefazolin was administered as routine antibiotic prophylaxis before surgery and continued for 24 h postoperatively. Postoperative analgesia consisted of intravenous paracetamol (1 g four times daily). Intravenous dexketoprofen was administered twice daily in patients without contraindications, including renal impairment. Unless contraindicated, all patients received low-molecular-weight heparin (enoxaparin, 40 mg once daily) for postoperative thromboprophylaxis. Patients treated with HA were mobilized with full weight-bearing to the extent they could tolerate it, while those treated with PFN followed a partial weight-bearing protocol. During the early postoperative rehabilitation period, all patients were mobilized using a walker. Data regarding mobilization status were obtained from electronic medical records.

Perioperative blood transfusion was considered in patients with a hemoglobin concentration < 8 g/dL who had symptoms attributable to anemia or persistent postoperative hemoglobin decline. During the hospital stay, hemoglobin levels, complete blood counts, serum biochemical parameters, and coagulation profiles were routinely monitored. Decisions regarding transfusions were based not on a single laboratory threshold value but on the patient’s overall clinical condition. When indicated, red blood cell suspensions were generally administered in two units.

### 2.5. Statistical Analysis

Statistical analyses were performed using IBM SPSS Statistics for Windows, Version 22.0 (IBM Corp., Armonk, NY, USA). Categorical variables are presented as frequencies and percentages, whereas continuous variables are presented as mean ± standard deviation or median (interquartile range), as appropriate according to their distribution.

The distribution of continuous variables was assessed using the Kolmogorov–Smirnov test together with visual inspection of histograms. Normally distributed continuous variables were compared using the independent-samples Student’s *t*-test, whereas non-normally distributed variables were compared using the Mann–Whitney *U* test. Categorical variables were compared using the chi-square test or Fisher’s exact test, as appropriate.

Separate univariate binary logistic regression analyses were conducted to evaluate the relationship between individual variables and POD. To enhance the interpretability of the estimated odds ratios, continuous variables were modeled using clinically meaningful units of increase. Due to its pronounced right-skewed distribution, the CAR was log_2_-transformed prior to regression analysis; thus, the reported odds ratio reflects the change in the likelihood of POD associated with a twofold increase in the CAR value.

A predefined multivariate binary logistic regression model was constructed to identify factors independently associated with POD. The demographic, clinical, perioperative, and inflammatory variables included in the model were selected based on their clinical significance and the existing literature, rather than using automated variable selection methods. Surgical procedure (hemiarthroplasty versus proximal femoral nailing) was included as the modeling variable because postoperative delirium occurred in both treatment groups. According to the institutional treatment protocol, the surgical approach was determined entirely by the type of fracture: displaced femoral neck fractures were treated with hemiarthroplasty, while intertrochanteric fractures were treated with proximal femoral nailing. Therefore, simultaneous inclusion of fracture type and surgical procedure would have introduced perfect collinearity. Fracture type was consequently not entered as a separate variable in the multivariable model. To avoid multicollinearity, CRP, albumin, and CAR were not entered simultaneously into the same model; the log_2_-transformed CAR was selected as the composite inflammatory marker. Multivariate linear relationships were assessed using variance inflation factors (VIF). The model’s discriminatory power was evaluated using the area under the receiver operating characteristic (ROC) curve (AUC); internal validation was performed using 1000 bootstrap resamples with an optimism correction. Model calibration was assessed using the Hosmer–Lemeshow goodness-of-fit test and calibration plots. Overall model performance was also evaluated using the Brier score and Nagelkerke R^2^. Results were presented as odds ratios (OR) along with 95% confidence intervals (CI). All statistical tests were two-sided, and *p* < 0.05 was considered statistically significant.

## 3. Results

### 3.1. Patient Characteristics

A total of 962 patients were included in the study, of whom 112 (11.6%) developed POD. The median time to POD onset was 2 postoperative days (interquartile range [IQR], 1–3 days). The patient selection flowchart is shown in Figure 1.

The median age of the entire cohort was 80 years (range, 50–109 years), and 591 patients (61.4%) were female. The median age was similar between patients with and without POD (80 [74–85] vs. 80 [72–87] years, *p* = 0.992). Likewise, no significant differences were observed across the predefined age categories (Table 1).

Patients who developed POD were more likely to be male than those without POD (50.9% vs. 36.9%, *p* = 0.004). Hemiarthroplasty was performed more frequently in the POD group, whereas proximal femoral nailing predominated among patients without POD (*p* < 0.001). Patients with POD also more frequently received blood transfusions (65.2% vs. 45.1%, *p* < 0.001), underwent general anesthesia (29.5% vs. 14.8%, *p* < 0.001), and had higher ASA physical status classifications (*p* < 0.001) (Table 1).

The median time from hospital admission to surgery was significantly longer in patients with POD than in those without POD (26 vs. 13 h, *p* < 0.001). Operative time was modestly longer in the POD group (75 vs. 68 min, *p* = 0.043). Both preoperative and postoperative mobilization were less frequent among patients who developed POD (both *p* < 0.001). Perioperative complications occurred markedly more frequently in the POD group (36.6% vs. 7.8%, *p* < 0.001). Pulmonary embolism (8.0% vs. 2.7%, *p* = 0.008) and deep vein thrombosis (5.4% vs. 1.5%, *p* = 0.017) were also significantly more frequent in the POD group (Table 1).

### 3.2. Laboratory Findings

Preoperative laboratory parameters according to the presence or absence of postoperative delirium are presented in Table 2. In patients who developed POD, preoperative hemoglobin levels were significantly lower (11.70 vs. 12.40 g/dL; *p* < 0.001) and international normalized ratio (INR) values were higher (*p* = 0.007) compared to patients who did not develop POD. In terms of biochemical parameters, calcium levels were lower in the POD group (*p* < 0.001), while urea (*p* < 0.001), creatinine (*p* = 0.017), and total bilirubin concentrations were higher (*p* < 0.001). No significant differences were observed between the groups in terms of white blood cell and platelet counts; sodium, potassium, and chloride levels; aspartate aminotransferase, alanine aminotransferase, and venous blood gas parameters (all *p* > 0.05).

Regarding inflammatory markers, patients who developed POD had significantly higher preoperative C-reactive protein (CRP) levels (25.75 vs. 12.20 mg/L, *p* < 0.001), erythrocyte sedimentation rate (ESR) (25.0 vs. 22.0 mm/h, *p* = 0.008), and C-reactive protein-to-albumin ratio (CAR) (0.76 vs. 0.33, *p* < 0.001), whereas serum albumin levels were significantly lower (33.0 vs. 36.0 g/L, *p* < 0.001) (Table 2).

### 3.3. Univariable Logistic Regression Analysis

Univariable logistic regression analyses were performed to assess the association between individual clinical and laboratory variables and postoperative delirium (POD) (Table 3).

Male sex, hemiarthroplasty, blood transfusion, general anesthesia, higher ASA physical status classification, longer time from hospital admission to surgery, and longer operative time were each associated with increased odds of POD in the univariable analyses. Several laboratory parameters were also significantly associated with POD, including lower hemoglobin, calcium, and albumin levels, as well as higher INR, urea, creatinine, total bilirubin, CRP, ESR, and CAR values (all *p* < 0.05).

Among the inflammatory biomarkers evaluated, the strongest univariate association was found for the log_2_-transformed CAR. A twofold increase in the CAR value was associated with a 46.4% increase in the likelihood of POD (OR: 1.464; 95% CI: 1.307–1.639; *p* < 0.001) (Table 3).

### 3.4. Multivariable Logistic Regression Analysis

Variables selected based on clinical significance and the results of univariate analysis were simultaneously included in a predefined multivariate logistic regression model. To prevent multicollinearity that could arise from the strong correlation between CRP, albumin, and the CAR, only the log_2_-transformed CAR was included in the model as a composite inflammatory marker.

After adjustment for potential confounders, male sex (OR 1.567, 95% CI 1.007–2.438; *p* = 0.046), hemiarthroplasty (OR 1.931, 95% CI 1.213–3.075; *p* = 0.006), blood transfusion (OR 1.870, 95% CI 1.178–2.971; *p* = 0.008), general anesthesia (OR 1.829, 95% CI 1.093–3.061; *p* = 0.021), higher ASA physical status classification (OR 2.154, 95% CI 1.346–3.446; *p* = 0.001), longer time from hospital admission to surgery (OR 1.250 per 24 h increase, 95% CI 1.103–1.417; *p* < 0.001), and a higher log_2_-transformed CAR (OR 1.184 per doubling, 95% CI 1.037–1.352; *p* = 0.013) remained independently associated with POD. In contrast, age, BMI, the presence of comorbidities, and operative time were not found to be independently associated with POD (Table 4).

### 3.5. Model Performance

The predictive performance of the final multivariate model is summarized in Table 5. The model demonstrated acceptable discriminatory power, with an area under the receiver operating characteristic (ROC) curve (AUC) of 0.764 (95% CI: 0.708–0.809). Following internal validation performed using 1000 bootstrap resamples, the optimism-corrected AUC was calculated as 0.744.

**Figure 2:**
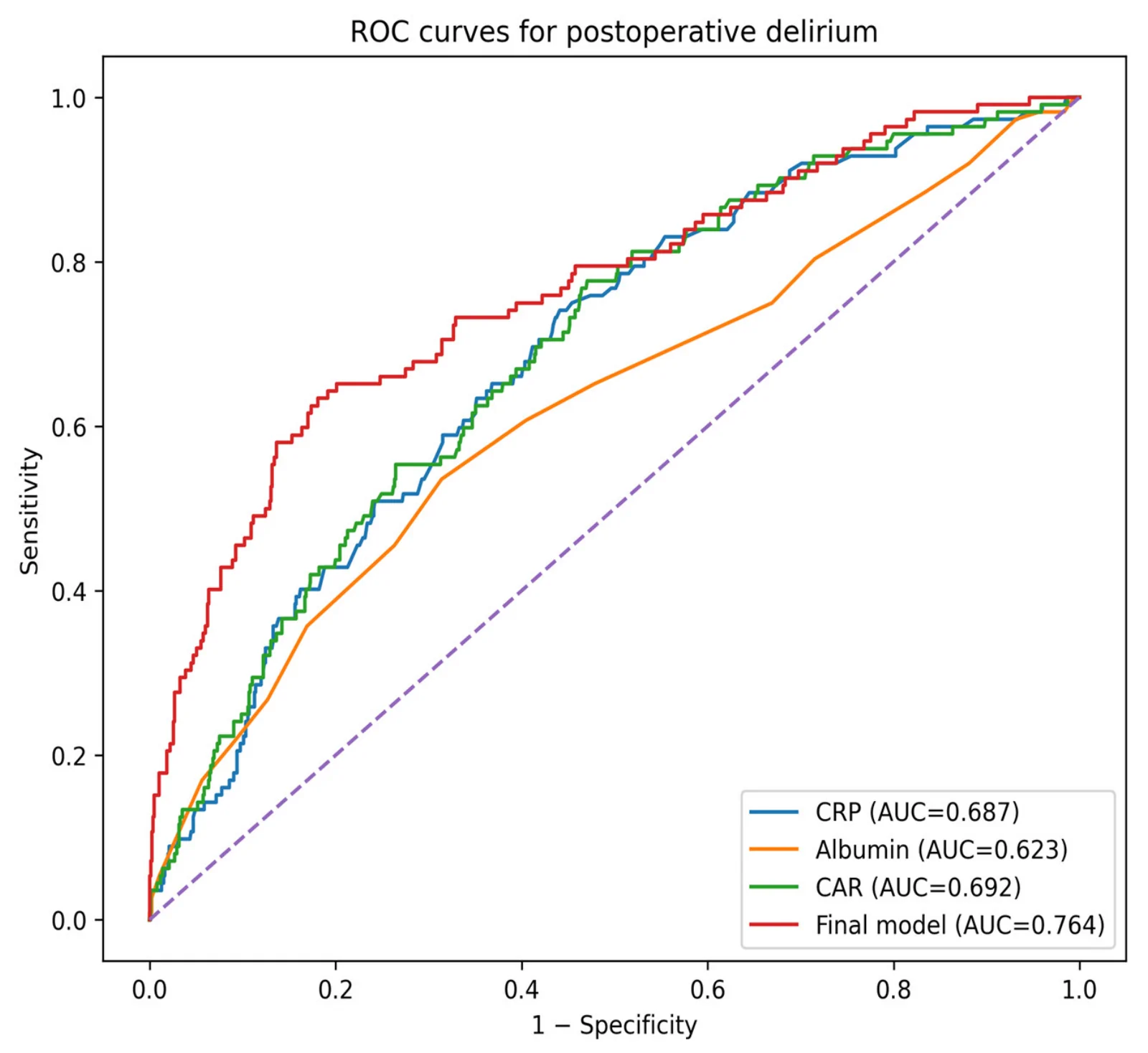
Receiver operating characteristic (ROC) curves for the final multivariable prediction model. The dotted line represents perfect calibration, where the predicted probabilities equal the observed proportions.

**Figure 3:**
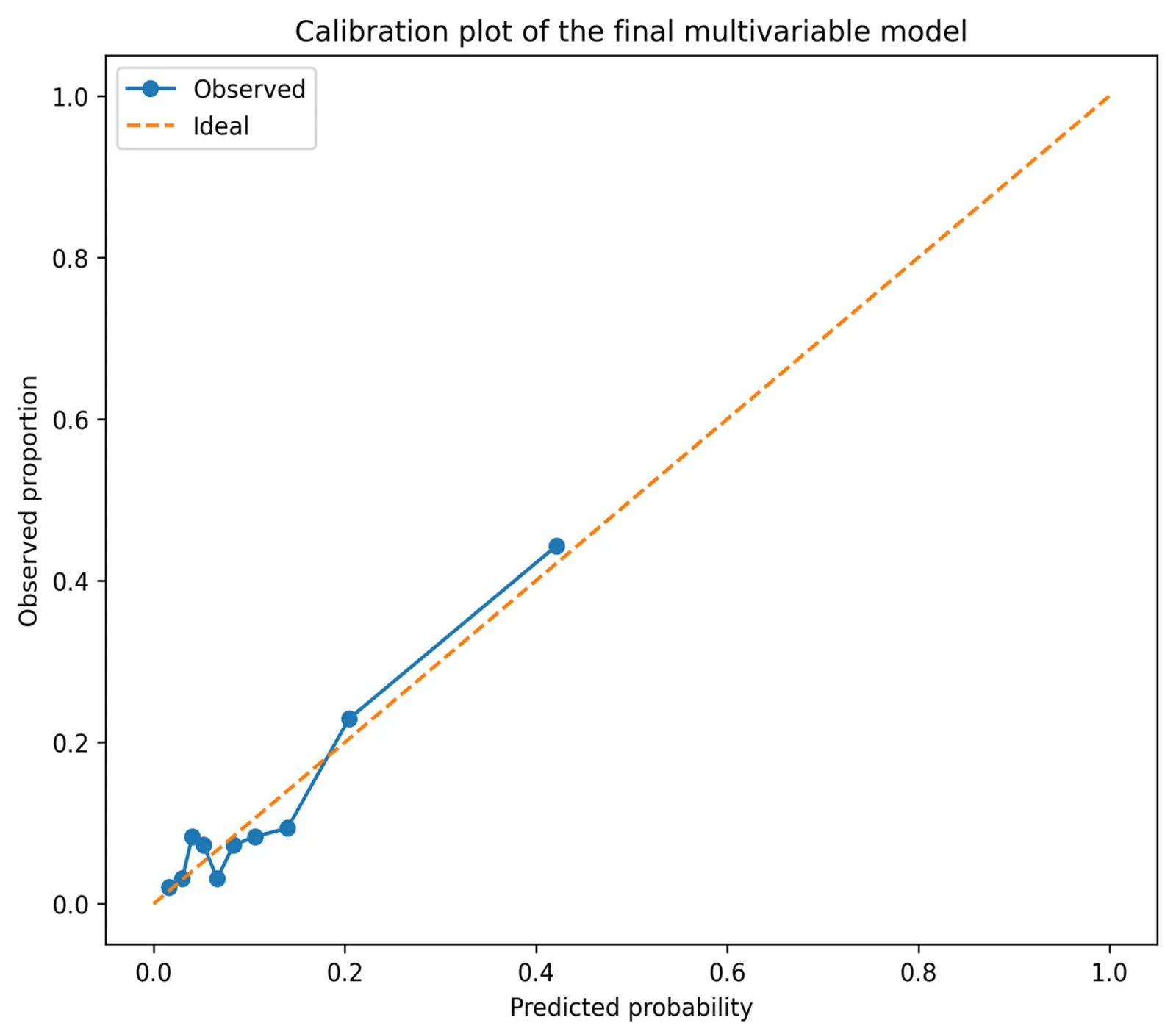
Calibration plot of the final multivariable prediction model.

Model calibration was satisfactory according to the Hosmer–Lemeshow goodness-of-fit test (χ^2^ = 10.332, *p* = 0.242). Overall predictive performance was supported by a Brier score of 0.086 and a Nagelkerke R^2^ of 0.218. Receiver operating characteristic curves are shown in Figure 2, and the calibration plot is presented in Figure 3.

## 4. Discussion

In this retrospective cohort study, we investigated the clinical and laboratory factors associated with POD following hip fracture surgery. The principal finding was that a higher preoperative CAR remained independently associated with POD after adjustment for demographic, clinical, and perioperative variables. In addition, male sex, hemiarthroplasty, higher ASA physical status classification, longer time from hospital admission to surgery, general anesthesia, and perioperative blood transfusion were independently associated with POD.

The overall incidence of POD in our cohort was 11.6%, which is within the lower range of the 13–55% incidence reported in previous studies of hip fracture surgery [16]. Variability among studies probably reflects differences in patient characteristics, delirium ascertainment methods, perioperative management protocols, and study design. In our institution, delirium was identified when clinically suspected and subsequently confirmed by a consultant psychiatrist according to DSM-5 criteria. Consequently, hypoactive delirium may have been underrecognized, which could have contributed to a lower observed incidence.

Increasing evidence suggests that systemic inflammation plays a central role in the pathogenesis of postoperative delirium. Surgical trauma induces a systemic inflammatory response that may increase blood–brain barrier permeability, activate microglia, and promote neuroinflammation, ultimately resulting in acute cerebral dysfunction [19,20,21,22]. Accordingly, numerous inflammatory biomarkers, including CRP, neutrophil-to-lymphocyte ratio, platelet-to-lymphocyte ratio, and composite inflammatory indices, have been evaluated as potential markers associated with POD [23,24,25,26].

Among these biomarkers, the CAR has attracted increasing attention because it integrates both systemic inflammatory activity and nutritional status into a single readily available parameter [27]. Zhang et al. reported that elevated CAR was associated with postoperative delirium after total knee arthroplasty [28], whereas Kim et al. demonstrated an independent association between higher preoperative CAR values and POD in elderly patients undergoing hip fracture surgery [29]. Consistent with these observations, patients who developed POD in our cohort had significantly higher preoperative CAR values, and this association remained significant after multivariable adjustment. Because CAR combines two routinely measured laboratory parameters without additional cost, it may represent a practical marker for identifying patients at increased risk of POD during preoperative assessment. Nevertheless, the observed effect size was modest. Therefore, CAR should be considered an adjunctive risk marker rather than a standalone predictive tool. Prospective multicenter studies are required to determine its incremental predictive value when combined with established clinical risk factors. Body mass index was associated with postoperative delirium in the univariable analysis but did not remain independently associated after multivariable adjustment. This finding suggests that BMI alone may not independently influence delirium risk and that its apparent association is likely mediated through accompanying clinical factors such as overall physiological status, nutritional reserve, or comorbidity burden. Lower preoperative hemoglobin levels were associated with POD in the univariable analysis. This finding may reflect reduced physiological reserve or greater preoperative illness burden. However, hemoglobin did not remain independently associated with POD after adjustment for other clinical variables, suggesting that its apparent effect may largely reflect overall patient condition rather than an independent contribution to delirium risk.

Unlike many previous studies, this analysis found that age was not independently associated with postoperative delirium. Advanced age is generally considered one of the strongest predictors of postoperative delirium following hip fracture surgery [31,32,33,34,35,36,37,38,39]. However, after adjusting for other demographic and perioperative variables, age did not retain its statistical significance in our cohort. This finding may be attributed to the relatively homogeneous age distribution in the study population, the influence of other clinical variables such as the ASA physical status classification and surgical delay, and the lack of routine use of standardized delirium screening. This situation may have led to insufficient recognition of cases of hypoactive delirium, particularly among the oldest patients. For this reason, the fact that the study was unable to demonstrate an independent association between age and postoperative delirium should be interpreted with caution and should not be regarded as a finding that contradicts the extensive evidence in the current literature.

Patients treated with hemiarthroplasty exhibited a higher incidence of POD than those treated with proximal femoral nailing, and hemiarthroplasty remained independently associated with POD after multivariable adjustment. However, this finding should not be interpreted as evidence that hemiarthroplasty itself causes postoperative delirium. In the treatment protocol of our study, the surgical method was determined based on the type of fracture; femoral neck fractures were treated with hemiarthroplasty, while intertrochanteric fractures were treated with proximal femoral nailing. Therefore, the observed association most likely reflects confounding by indication and underlying case-mix differences related to fracture characteristics and patient selection, rather than a direct effect of the surgical procedure itself. Accordingly, this finding should not be interpreted as evidence favoring one surgical procedure over the other with respect to delirium risk.

General anesthesia was independently associated with POD in the adjusted model. Although the influence of anesthesia type on postoperative delirium remains controversial, several studies have suggested that general anesthesia may be associated with greater postoperative cognitive disturbance than neuraxial techniques in selected patient populations, whereas others have reported no meaningful differences [40,41,42,43]. Since the type of anesthesia was not randomly assigned in this retrospective study, patients who received general anesthesia may have systematically differed from those who received spinal anesthesia in terms of initial clinical condition, suitability for anesthesia, or perioperative risk. Accordingly, residual confounding by indication and patient selection cannot be excluded, and the observed association should not be interpreted as evidence that general anesthesia itself increases the risk of postoperative delirium.

Longer time from hospital admission to surgery was also independently associated with POD. Delayed surgery may prolong pain, immobilization, sleep disturbance, and exposure to physiological stress, all of which have been proposed as contributing factors to delirium development [44,45]. Our findings further support current guidelines and recommendations that advise performing surgical treatment without delay whenever clinically feasible.

Although operative time was not independently associated with postoperative delirium after adjustment, it was retained in the prespecified multivariable model because operative duration is a clinically relevant perioperative variable that has been associated with postoperative delirium in previous studies [17,18]. Since our model was constructed on the basis of clinical relevance rather than automated variable selection, clinically important variables were retained regardless of their individual statistical significance to provide more robust estimates for the remaining predictors.

Patients who received perioperative blood transfusions had a higher likelihood of developing POD after adjustment for the variables included in the model. This finding should not be interpreted as evidence that blood transfusion itself has a harmful causal effect. Rather, perioperative blood transfusion is more likely to represent a marker of illness severity, reflecting greater blood loss, perioperative anemia, increased surgical complexity, or reduced physiological reserve rather than an independent causal determinant of postoperative delirium. Moreover, because the temporal relationship between transfusion and delirium onset could not be reliably established in this retrospective cohort, reverse causation and residual confounding cannot be excluded [46,47,48].

Although operative time was associated with postoperative delirium in the univariable analysis, this association was no longer significant after multivariable adjustment. Previous studies have also evaluated differences in clinical and cognitive outcomes between hemiarthroplasty and proximal femoral nailing, as well as risk factors associated with postoperative delirium after hip surgery[49,50,51]. No evidence of problematic multicollinearity was identified based on variance inflation factor analyses. Therefore, attenuation of the association after adjustment is more likely attributable to shared clinical characteristics related to fracture type, surgical complexity, and perioperative management than to statistical multicollinearity. Patients who received perioperative blood transfusions had a higher likelihood of developing POD after adjustment for the variables included in the model [52,53,54].

### Strengths and Limitations

This study has several strengths that should be taken into account when interpreting the findings and evaluating them in a clinical context. It included a relatively large cohort of 962 patients, all delirium diagnoses were confirmed by consultant psychiatrists according to DSM-5 criteria, and perioperative management was relatively standardized because all procedures were performed by the same orthopedic team. Furthermore, the study evaluated CAR, an inexpensive and universally available biomarker, using a prespecified multivariable model with bootstrap internal validation, thereby strengthening the robustness and generalizability of the statistical findings.

Nevertheless, several limitations should be acknowledged. First, the retrospective single-center design precludes causal inference. Second, since standardized routine delirium screening tools such as the Confusion Assessment Method were not used, cases of hypoactive delirium in particular may not have been adequately identified. Third, since the surgical method was determined based on the type of fracture, residual confounding by indication cannot be completely ruled out, despite multivariate adjustment. Fourth, due to the study’s retrospective design, the temporal relationship between perioperative complications and the onset of postoperative delirium could not be reliably determined. Therefore, perioperative complications should be interpreted as factors associated with postoperative delirium rather than as confirmed antecedent events. Fifth, some potentially important variables—such as baseline cognitive status, frailty, standardized nutritional assessment, educational level, and use of psychoactive medications—were either missing or not recorded; therefore, they could not be included in the regression model. Furthermore, comorbidity was assessed as a binary (present/absent) variable rather than a validated comorbidity index, which may have reduced the level of detail in the adjustment for comorbidity. Finally, although the predictive model has undergone bootstrap-based internal validation, there is no independent external validation cohort. Therefore, external validation in prospective, multicenter studies is required before the model can be used more widely in clinical practice.

## 5. Conclusions

In this retrospective cohort study, a higher preoperative CAR remained independently associated with postoperative delirium after hip fracture surgery, even after adjustment for established demographic and perioperative risk factors. Male sex, hemiarthroplasty, general anesthesia, higher ASA physical status, longer time from hospital admission to surgery, and perioperative blood transfusion were also independently associated with POD. Together, these findings suggest that the preoperative CAR may contribute to perioperative risk stratification when interpreted alongside established clinical risk factors rather than as a standalone predictive marker. Prospective multicenter studies incorporating standardized delirium screening and external validation are required before routine clinical implementation can be recommended.

## Figures and Tables

**Table 1:** Demographic and clinical characteristics of patients with and without postoperative delirium.

Variable	Total (n = 962)	Without POD (n = 850)	With POD (n = 112)	*p* Value
Age, years	80 (71–90)	80 (71–88)	80 (72–90)	0.992
**Age group, n (%)**				
<65 years	101 (10.5%)	89 (10.5%)	12 (10.7%)	
65–74 years	208 (21.6%)	190 (22.4%)	18 (16.1%)	
75–84 years	332 (34.5%)	286 (33.6%)	46 (41.1%)	
≥85 years	321 (33.4%)	285 (33.5%)	36 (32.1%)	
Male sex, n (%)	371 (38.6%)	314 (36.9%)	57 (50.9%)	**0.004**
Fracture side, right, n (%)	483 (50.2%)	429 (50.5%)	54 (48.2%)	0.653
**Surgical procedure, n (%)**				**<0.001**
Proximal femoral nailing (PFN)	630 (65.5%)	579 (68.1%)	51 (45.5%)	
Hemiarthroplasty (HA)	332 (34.5%)	271 (31.9%)	61 (54.5%)	
BMI, kg/m^2^	26.1 (23.0–30.0)	27.8 (24.0–29.0)	25.4 (23.0–30.0)	**0.036**
Any comorbidity, n (%)	914 (95.0%)	803 (94.5%)	111 (99.1%)	**0.034**
Blood transfusion, n (%)	456 (47.4%)	383 (45.1%)	73 (65.2%)	**<0.001**
**Anesthesia type, n (%)**				**<0.001**
Spinal	803 (83.5%)	724 (85.2%)	79 (70.5%)	
General	159 (16.5%)	126 (14.8%)	33 (29.5%)	
**ASA physical status, n (%)**				**<0.001**
ASA II	46 (4.8%)	46 (5.4%)	0 (0.0%)	
ASA III	693 (72.0%)	636 (74.8%)	57 (50.9%)	
ASA IV	223 (23.2%)	168 (19.8%)	55 (49.1%)	
Time from admission to surgery, h	14 (6–28)	13 (6–26)	26 (9–72)	**<0.001**
Operative time, min	70 (56–85)	68 (55–82)	75 (60–86)	**0.043**
Preoperative mobilization, n (%)	875 (91.0%)	796 (93.6%)	79 (70.5%)	**<0.001**
Postoperative mobilization, n (%)	870 (90.4%)	791 (93.1%)	79 (70.5%)	**<0.001**
Perioperative complication, n (%)	107 (11.1%)	66 (7.8%)	41 (36.6%)	**<0.001**
Pulmonary embolism, n (%)	32 (3.3%)	23 (2.7%)	9 (8.0%)	**0.008**
Deep vein thrombosis, n (%)	19 (2.0%)	13 (1.5%)	6 (5.4%)	**0.017**

Note. Continuous variables are presented as median (interquartile range) and were compared using the Mann–Whitney U test. Categorical variables are presented as n (%) and were compared using the chi-square test or Fisher’s exact test, as appropriate. Age categories are presented for descriptive purposes only. Age was entered into the regression analyses as a continuous variable. POD, postoperative delirium; PFN, proximal femoral nailing; HA, hemiarthroplasty; BMI, body mass index; ASA, American Society of Anesthesiologists. Bold values indicate statistical significance (*p* < 0.05).

**Table 2:** Comparison of preoperative laboratory parameters.

Variable	Total (n = 962)	Without POD (n = 850)	With POD (n = 112)	*p* Value
**Hematological parameters**				
Hb (g/dL)	12.30 (11.00–13.60)	12.40 (11.10–13.67)	11.70 (10.20–12.95)	**<0.001**
WBC (×10^3^/µL)	10.10 (7.90–12.70)	10.20 (7.90–12.80)	9.65 (7.78–11.75)	0.204
PLT (×10^3^/µL)	231.0 (187.0–287.8)	230.5 (186.2–284.0)	235.0 (196.5–306.2)	0.157
INR	1.08 (1.00–1.10)	1.07 (1.00–1.10)	1.10 (1.03–1.19)	**0.007**
**Biochemical parameters**				
Na (mmol/L)	138.0 (136.0–140.0)	138.0 (136.0–140.0)	138.0 (135.8–140.0)	0.960
K (mmol/L)	4.30 (4.00–4.60)	4.30 (4.00–4.60)	4.27 (3.90–4.50)	0.272
Cl (mmol/L)	104.0 (102.0–107.0)	104.0 (102.0–107.0)	104.0 (101.8–107.0)	0.840
Ca (mg/dL)	9.00 (8.60–9.30)	9.00 (8.62–9.30)	8.90 (8.47–9.10)	**<0.001**
AST (U/L)	22.0 (18.0–28.0)	22.0 (18.0–28.0)	23.0 (18.0–29.2)	0.132
ALT (U/L)	14.0 (10.0–19.0)	14.0 (10.0–19.0)	14.0 (11.0–21.2)	0.130
Urea (mg/dL)	47.0 (36.0–64.0)	46.0 (36.0–63.0)	54.0 (41.0–75.0)	**<0.001**
Creatinine (mg/dL)	0.90 (0.70–1.10)	0.83 (0.70–1.10)	0.90 (0.70–1.38)	**0.017**
Total bilirubin (mg/dL)	0.60 (0.50–0.70)	0.60 (0.50–0.70)	0.70 (0.50–0.80)	**<0.001**
**Venous blood gas analysis**				
PvO_2_ (mmHg)	44.0 (41.4–49.0)	44.0 (41.5–49.0)	44.0 (41.1–52.0)	0.400
PvCO_2_ (mmHg)	36.7 (34.0–38.0)	36.7 (34.0–38.0)	36.5 (33.0–38.5)	0.599
Lactate (mmol/L)	2.10 (1.50–2.50)	2.10 (1.50–2.50)	2.05 (1.40–2.52)	0.384
**Inflammatory parameters**				
CRP (mg/L)	12.50 (4.80–27.90)	12.20 (4.70–25.00)	25.75 (12.57–65.12)	**<0.001**
ESR (mm/h)	22.0 (16.0–35.0)	22.0 (16.0–34.8)	25.0 (18.0–38.2)	**0.008**
Albumin (g/L)	36.0 (32.0–38.0)	36.0 (32.0–38.0)	33.0 (30.0–36.2)	**<0.001**
CAR	0.37 (0.14–0.80)	0.33 (0.13–0.74)	0.76 (0.37–2.02)	**<0.001**

Note. Continuous variables are presented as median (interquartile range) and were compared using the Mann–Whitney U test. Venous blood gas values were obtained from venous samples and are reported as PvO_2_ and PvCO_2_. CAR was calculated as CRP (mg/L) divided by albumin (g/L) and is dimensionless. POD, postoperative delirium; Hb, hemoglobin; WBC, white blood cell count; PLT, platelet count; INR, international normalized ratio; AST, aspartate aminotransferase; ALT, alanine aminotransferase; CRP, C-reactive protein; ESR, erythrocyte sedimentation rate; CAR, C-reactive protein-to-albumin ratio.

**Table 3:** Univariable logistic regression analysis of factors associated with postoperative delirium.

Variable	Unadjusted OR	95% CI	*p* Value
**Demographic and clinical variables**			
Age, per 10-year increase	1.034	0.853–1.252	0.735
Male sex (vs. female)	1.769	1.191–2.628	**0.005**
Hemiarthroplasty (vs. PFN)	2.555	1.715–3.808	**<0.001**
BMI, per 5 kg/m^2^ increase	0.584	0.364–0.937	**0.026**
Any comorbidity (yes vs. no)	6.497	0.888–47.56	0.065
Blood transfusion (yes vs. no)	2.282	1.512–3.444	**<0.001**
General anesthesia (vs. spinal)	2.400	1.533–3.757	**<0.001**
ASA physical status, per 1-class increase	3.889	2.628–5.757	**<0.001**
Time from admission to surgery, per 24 h increase	1.432	1.273–1.611	**<0.001**
Operative time, per 10 min increase	1.088	1.009–1.174	**0.029**
**Hematological parameters**			
Hb, per 1 g/dL increase	0.851	0.769–0.942	**0.002**
WBC, per 1 × 10^3^/µL increase	0.986	0.935–1.040	0.603
PLT, per 50 × 10^3^/µL increase	1.033	0.942–1.132	0.493
INR, per 1-unit increase	3.200	1.335–7.669	**0.009**
**Biochemical parameters**			
Na, per 1 mmol/L increase	0.968	0.917–1.021	0.234
K, per 1 mmol/L increase	0.828	0.558–1.229	0.349
Cl, per 1 mmol/L increase	1.008	0.962–1.057	0.733
Ca, per 1 mg/dL increase	0.528	0.387–0.720	**<0.001**
AST, per 10 U/L increase	1.079	0.997–1.167	0.058
ALT, per 10 U/L increase	1.063	0.958–1.179	0.250
Urea, per 10 mg/dL increase	1.127	1.062–1.197	**<0.001**
Creatinine, per 1 mg/dL increase	1.361	1.133–1.634	**<0.001**
Total bilirubin, per 1 mg/dL increase	1.878	1.136–3.104	**0.014**
**Venous blood gas parameters**			
PvO_2_, per 10 mmHg increase	1.326	1.074–1.637	**0.009**
PvCO_2_, per 10 mmHg increase	0.953	0.571–1.588	0.853
Lactate, per 1 mmol/L increase	1.073	0.863–1.333	0.525
**Inflammatory parameters**			
CRP, per 10 mg/L increase	1.094	1.057–1.133	**<0.001**
ESR, per 10 mm/h increase	1.145	1.028–1.276	**0.014**
Albumin, per 1 g/L increase	0.885	0.842–0.931	**<0.001**
CAR, per doubling	1.464	1.307–1.639	**<0.001**

Note. ORs were estimated using separate univariable binary logistic regression models; all models included 962 patients and 112 POD events. Continuous variables were scaled to clinically interpretable increments as specified in the variable labels. CAR was log_2_-transformed; therefore, its OR represents the change in the odds of POD associated with a doubling of CAR. ASA physical status was modeled as an ordinal variable. Reference categories were female sex, PFN, no comorbidity, no transfusion, and spinal anesthesia. Variables included in the multivariable model were selected on the basis of both clinical relevance and the univariable analyses, as prespecified in the Section 2.5. OR, odds ratio; CI, confidence interval; POD, postoperative delirium; PFN, proximal femoral nailing; HA, hemiarthroplasty; BMI, body mass index; CAR, C-reactive protein-to-albumin ratio.

**Table 4:** Multivariable logistic regression analysis of factors independently associated with postoperative delirium.

Variable	Adjusted OR	95% CI	*p* Value
Age, per 10-year increase	0.900	0.711–1.138	0.378
Male sex (vs. female)	1.567	1.007–2.438	**0.046**
Hemiarthroplasty (vs. PFN)	1.931	1.213–3.075	**0.006**
BMI, per 5 kg/m^2^ increase	0.698	0.412–1.181	0.180
Any comorbidity (yes vs. no)	2.232	0.285–17.45	0.444
Blood transfusion (yes vs. no)	1.870	1.178–2.971	**0.008**
General anesthesia (vs. spinal)	1.829	1.093–3.061	**0.021**
ASA physical status, per 1-class increase	2.154	1.346–3.446	**0.001**
Time from admission to surgery, per 24 h increase	1.250	1.103–1.417	**<0.001**
Operative time, per 10 min increase	0.998	0.913–1.090	0.963
CAR, per doubling	1.184	1.037–1.352	**0.013**

Note. The prespecified model included 962 patients and 112 POD events. All listed variables were entered simultaneously; no stepwise selection procedure was used. CAR was log_2_-transformed; therefore, its OR represents the change in the odds of POD associated with a doubling of CAR. No problematic multicollinearity was detected (maximum VIF = 1.48). Reference categories were female sex, PFN, no comorbidity, no transfusion, and spinal anesthesia. OR, odds ratio; CI, confidence interval; POD, postoperative delirium; PFN, proximal femoral nailing; HA, hemiarthroplasty; BMI, body mass index; ASA, American Society of Anesthesiologists; CAR, C-reactive protein-to-albumin ratio.

**Table 5:** Diagnostic performance and internal validation of the final multivariable prediction model for postoperative delirium.

Metric	Value
**Patients included**	**962**
**POD events**	**112**
AUC (apparent)	0.764
95% CI	0.708–0.809
Optimism-corrected AUC	0.744
Hosmer–Lemeshow χ^2^	10.332
Hosmer–Lemeshow *p*	0.242
Brier score	0.086
Nagelkerke R^2^	0.218
Maximum VIF	1.48

Note. The final multivariable model included 962 patients, of whom 112 (11.6%) developed postoperative delirium. Internal validation was performed using 1000 bootstrap resamples. The optimism-corrected AUC was estimated from 912 valid bootstrap samples. Calibration was assessed using the Hosmer–Lemeshow good-ness-of-fit test. AUC, area under the receiver operating characteristic curve; VIF, variance inflation factor.

## Data Availability

The datasets generated and/or analyzed during the current study are available from the corresponding author on reasonable request.

## Abbreviations

ASA	American Society of Anesthesiologists
AST	Aspartate aminotransferase
BMI	Body mass index
CAM	Confusion Assessment Method
CAR	C-reactive protein-to-albumin ratio
CKD	Chronic kidney disease
COPD	Chronic obstructive pulmonary disease
CRP	C-reactive protein
DM	Diabetes mellitus
DSM-5	Diagnostic and Statistical Manual of Mental Disorders, Fifth Edition
DVT	Deep vein thrombosis
ESR	Erythrocyte sedimentation rate
Hb	Hemoglobin
HT	Hypertension
INR	International normalized ratio
PE	Pulmonary embolism
PFN	Proximal femoral nailing
POD	Postoperative delirium
PvCO_2_	Venous partial pressure of carbon dioxide
PvO_2_	Venous partial pressure of oxygen
WBC	White blood cell count
